# A phase I study of irinotecan and pegylated liposomal doxorubicin in recurrent ovarian cancer (Tohoku Gynecologic Cancer Unit 104 study)

**DOI:** 10.1007/s00280-014-2418-8

**Published:** 2014-03-01

**Authors:** Tadahiro Shoji, Eriko Takatori, Yoshitaka Kaido, Hideo Omi, Yoshihito Yokoyama, Hideki Mizunuma, Michiko Kaiho, Takeo Otsuki, Tadao Takano, Nobuo Yaegashi, Hiroshi Nishiyama, Keiya Fujimori, Toru Sugiyama

**Affiliations:** 1 Department of Obstetrics and Gynecology, Iwate Medical University School of Medicine, 19-1 Uchimaru, Morioka, 020-8505 Japan; 2 Department of Obstetrics and Gynecology, Hirosaki University School of Medicine, 53 Honcho, Hirosaki, 036-8563 Japan; 3 Department of Obstetrics and Gynecology, Tohoku University Graduate School of Medicine, 1-1 Seiryouchou, Sendai, 980-8574 Japan; 4 Department of Obstetrics and Gynecology, Fukushima Medical University School of Medicine, 1 Hikarigaoka, Fukushima, 960-1295 Japan

**Keywords:** Recurrent ovarian cancer, Chemotherapy, CPT-11, PLD

## Abstract

**Purpose:**

A phase I clinical study was conducted to determine the maximum tolerated dose (MTD) and the recommended dose (RD) of irinotecan hydrochloride (CPT-11) in CPT-11/pegylated liposomal doxorubicin (PLD) combination therapy, a novel treatment regimen for platinum- and taxane-resistant recurrent ovarian cancer.

**Methods:**

Pegylated liposomal doxorubicin was administered intravenously on day 3 at a fixed dose of 30 mg/m^2^. CPT-11 was administered intravenously on days 1 and 15, at a dose of 50 mg/m^2^ on both days. One course of chemotherapy was 28 days, and patients were given a maximum of six courses, with the CPT-11 dose being increased in increments of 10 mg/m^2^ (level 1, 50 mg/m^2^; level 2, 60 mg/m^2^; level 3, 70 mg/m^2^; level 4, 80 mg/m^2^) to determine MTD and RD.

**Results:**

During the period from April 2010 to March 2013, three patients were enrolled for each level. In the first course, no dose-limiting toxicity occurred in any of the patients. Grade 4 neutropenia was observed in two of three patients at level 4. At level 4, the antitumor effect was a partial response (PR) in two of the three patients and stable disease (SD) in one. At level 3, one of the three patients showed PR and two had SD. At level 4, the start of the next course was postponed in two of three patients. In addition, one patient at level 4 experienced hemotoxicity that met the criteria for dose reduction in the next course. The above results suggested that administration of CPT-11 at dose level 5 (90 mg/m^2^) would result in more patients with severe neutropenia and in more patients requiring postponement of the next course or a dose reduction. Based on the above, the RD of CPT-11 was determined to be 80 mg/m^2^.

**Conclusions:**

The results suggest that CPT-11/PLD combination therapy for recurrent ovarian cancer is a useful treatment method with a high response rate and manageable adverse reactions. In the future phase II study, the safety and efficacy of this therapy will be assessed at 80 mg/m^2^ of CPT-11 and 30 mg/m^2^ of PLD.

## Introduction

The standard initial chemotherapy for advanced ovarian cancer is paclitaxel plus carboplatin (TC) combination therapy [1–3]. However, no treatment regimen for second-line chemotherapy has yet been established against recurrence after TC therapy. Various attempts are currently being made using, as criteria, the type of recurrence and the period from the last treatment until recurrence. Since recurrent ovarian cancer with a treatment-free interval (the period from the end of the initial chemotherapy until recurrence) of <6 months is considered to be platinum resistant, it will be essential to select drugs not showing cross-resistance with the initial therapy. In the United States and Europe, the type I DNA topoisomerase inhibitor topotecan [4], pegylated liposomal doxorubicin (PLD) [5], and gemcitabine [6] are used against platinum-resistant recurrent ovarian cancer. In a Japanese phase II study involving patients with ovarian cancer previously treated with chemotherapy including platinum-based agents, PLD was reported to achieve an overall response rate of 21.9 % (27.3 % [3/11] in the platinum-sensitive group and 21.0 % [13/62] in the platinum-resistant group) [7]. In a phase III non-inferiority study comparing PLD with topotecan, it was reported that in patients treated with PLD, the response rate was 19.7 %, median progression-free survival (PFS) was 16.1 weeks, and mean survival time (MST) was 60.0 weeks and, in patients with platinum-resistant tumors in particular, the response rate was 12.3 %, median PFS was 9.1 weeks, and MST was 35.6 weeks [8], suggesting that PLD would be a promising therapeutic agent for recurrent ovarian cancer. On the other hand, irinotecan hydrochloride (CPT-11), an anticancer agent developed in Japan, acts by inhibiting topoisomerase I. In a study in which CPT-11 (100 mg/m^2^) alone was administered to patients with platinum-resistant recurrent ovarian cancer, the response rate was 29 %, the tumor growth inhibition rate (complete response [CR] + partial response [PR] + not changed) was 61 %, median time to progression was 17 weeks, and MST was 8 months, exhibiting favorable results [9]. Sugiyama et al. [10] reported that CPT-11/cisplatin therapy was effective as second-line chemotherapy for recurrent ovarian cancer after treatment with a platinum agent, raising the expectation that CPT-11 may be effective against platinum- and taxane-resistant tumors.

Herein, we conducted a phase I clinical study to determine the maximum tolerated dose (MTD) and the recommended dose (RD) of CPT-11 in CPT-11/PLD combination therapy, a novel treatment regimen for platinum- and taxane-resistant recurrent ovarian cancer, with the aim of improving the outcomes of ovarian cancer patients.

## Subjects and methods

### Study population

Upon receiving approval from the intramural ethics committee of each study center, a multicenter clinical study was conducted in patients with recurrent ovarian cancer who met the following criteria and were enrolled in the study during the period from April 2010 to March 2013: (1) ovarian cancer confirmed by histological or cytological diagnosis, (2) recurrence less than 6 months after previous chemotherapy, (3) containing a measurable or evaluable lesion (including CA-125 level), (4) ECOG performance status (PS) 0–2, (5) 20–75-year old, (6) expected survival time of at least 2 months, (7) major organs remained functional (white blood cell count ≥3,000/mm^3^, neutrophil count ≥1,500/mm^3^, platelet count ≥10,0000/mm^3^, total bilirubin ≤1.5 mg/dL), and (8) informed consent provided. Exclusion criteria were (1) serious complication(s), (2) evident pulmonary fibrosis or interstitial pneumonitis, (3) pleural or cardiac effusion necessitating prompt local treatment, (4) brain metastasis requiring prompt treatment, (5) diarrhea (watery stool), (6) intestinal paralysis or intestinal obstruction, (7) active infection requiring treatment with antimicrobial agents, and (8) patients considered inappropriate as subjects by the physician in charge for any other reason.

### Protocol

Pegylated liposomal doxorubicin was administered intravenously at a fixed dose of 30 mg/m^2^ on day 3. CPT-11 was administered intravenously on days 1 and 15. One course of chemotherapy was 28 days, and as a general rule, patients were given at least 2 courses, 6 courses at the maximum.

#### Method for dose escalation

CPT-11 was started at level 1 (50 mg/m^2^) and then increased up to level 4 (80 mg/m^2^) (Table 1). A group of three patients were given the same dose level of CPT-11, and if no dose-limiting toxicity (DLT) was observed in any of them, the dose was increased to the next level. If DLT was observed in one of the three patients at the same level, three additional patients were treated at the same dose level, and if there was no observable DLT in at least three of the total six patients, the dose was increased to the next level. If DLT was observed in at least three of the total six patients, the dose was judged to be MTD. If DLT was observed in two of three patients at any level, this dose level was judged to be MTD. The dose that was 1 level below MTD was determined to be RD. DLT was defined as (1) grade 4 leukopenia or neutropenia lasting for at least 4 days, (2) grade 3 or higher leukopenia or neutropenia accompanied by pyrexia of ≥38 °C, (3) grade 4 or higher thrombocytopenia or thrombocytopenia requiring platelet transfusion, or (4) grade 3 or higher nonhematological toxicity (except nausea/vomiting, anorexia, and general malaise). Adverse events were evaluated according to NCI-CTCAE ver. 3, and MTD was determined during the first course.

**Table 1:** Dose escalation schema

	CPT-11 (mg/m^2^)	PLD (mg/m^2^)
Level 0	40	30
Level 1	50	30
Level 2	60	30
Level 3	70	30
Level 4	80	30

#### Criteria for changing dosing schedule

If any of the following applied, CPT-11 administration on day 15 was to be postponed and the drug was to be administered on day 22 upon confirming recovery from the condition: (1) white blood cell count ≤2,000/mm^3^, (2) neutrophil count ≤1,000/mm^3^, (3) platelet count ≤75,000/mm^3^, or (4) grade 1 or higher diarrhea. If recovery from the condition was not seen on day 22, the second CPT-11 administration was to be skipped (not to be administered on day 29). The criteria for proceeding to the second and subsequent courses were (1) white blood cell count ≥3,000/mm^3^, (2) neutrophil count ≥1,500/mm^3^, (3) platelet count ≥100,000/mm^3^, (4) total bilirubin ≤1.5 mg/dL, (5) diarrhea grade 0, and (6) grade 1 or lower hand-and-foot syndrome and stomatitis. If the patient met any of the above criteria, administration was to be performed after waiting for recovery for a maximum of 14 days. If recovery from these conditions was not seen after 14 days, the treatment was to be discontinued. If the severity of hand-and-foot syndrome or stomatitis remained at grade 2 or higher after a 14-day postponement, PLD on day 3 in the next course was to be skipped.

#### Criteria for dose reduction

The doses of CPT-11 and PLD in the next course were reduced according to the severity of adverse reactions that occurred in the previous course. If grade 4 leukopenia, grade 4 neutropenia, or grade 3 thrombocytopenia were observed in the previous course, CPT-11 was reduced by 10 mg/m^2^, and PLD by 7.5 mg/m^2^. If grade 2 or higher diarrhea, spasmodic abdominal pain, or watery stool were observed, the CPT-11 dose was reduced by 10 mg/m^2^. If grade 3 hand-and-foot syndrome or stomatitis was observed, the PLD dose was reduced by 7.5 mg/m^2^ regardless of whether or not these conditions improved before the start of the next course.

### Evaluation of antitumor effect

The antitumor effect was evaluated by imaging at the end of every two courses. For the evaluation of the antitumor effect, the best response rate was calculated according to the Response Evaluation Criteria in Solid Tumors (RECIST) guideline.

## Results

### Patient background characteristics

Table 2 shows the background characteristics of 12 patients enrolled in this study during the period from April 2010 through March 2013. All patients had been treated with taxane- or platinum-based agents as a part of the previous therapy.

**Table 2:** Patients characteristics (N=12)

Age	
Median	56
Range	40–65
PS	
0	11
1	1
FIOG stage	
I	2
II	1
III	8
IV	1
Histological type	
Serous	8
Mucinus	0
Clear cell	3
Endometrioid	1
Previous regimens	
1	4
2	3
3≤	5
Last regimen	
TC	8
TP	1
DP	1
CDDP/VP16	1
PTX	1

*TC* paclitaxel/carboplatin, *TP* paclitaxel/cisplatin, *DP* docetaxel/cisplatin, *CDDP* cisplatin, *PTX* paclitaxel

### Adverse events

Three patients were enrolled for each level, and none of them experienced DLT in the first course. No grade 3 or higher neutropenia was observed at level 1. Grade 4 leukopenia was observed in one patient each at level 2 and level 3, and in two patients at level 4. No grade 3 or higher thrombocytopenia was observed at level 1 or 2. At level 3, grade 3 thrombocytopenia was observed in one patient, and at level 4, two patients developed grade 2 thrombocytopenia, while no grade 3 or higher thrombocytopenia was observed. The only grade 2 or higher nonhematological toxicity was grade 2 hand-and-foot syndrome, which occurred in one patient at level 4 (Table 3).

**Table 3:** Toxicities

	Level l (*n* = 3)				Level 2 (*n* = 3)				Level 3 (*n* = 3)				Level 4 (*n* = 3)			
Toxicity grade	1	2	3	4	1	2	3	4	1	2	3	4	1	2	3	4
Hematological																
Leukopenia	0	3	0	0	0	2	1	0	0	2	1	0	0	2	1	0
Neutropenia	0	3	0	0	0	2	0	1	0	2	0	1	0	1	0	2
Thrombocytopenia	0	0	0	0	1	0	0	0	0	0	1	0	1	0	0	0
Anemia	3	0	0	0	2	1	0	0	1	1	0	0	2	0	0	0
Nonhematological																
Mucositis	1	0	0	0	1	0	0	0	0	0	0	0	3	0	0	0
Hand foot	1	0	0	0	1	0	0	0	0	0	0	0	1	1	0	0
Diarrhea	2	0	0	0	0	0	0	0	1	0	0	0	0	0	0	0
Nausea	2	0	0	0	1	0	0	0	1	0	0	0	1	0	0	0
Vomiting	2	0	0	0	0	0	0	0	0	0	0	0	1	0	0	0
Appetite loss	2	0	0	0	0	0	0	0	1	0	0	0	0	0	0	0

### Administration status

In total, 43 treatment courses were administered. Table 4 shows the status of postponement of the next course, dose skipping, and dose reduction in each patient. The start of the next course was postponed due to the lack of recovery from neutropenia in one patient each at levels 1, 3, and 4 and in two patients at level 2. All of these patients started the next course within 7 days without using granulocyte colony-stimulating factor. Postponement of the next course due to the lack of recovery from hand-and-foot syndrome occurred in one patient at level 4, but the next course was started within 7 days.

**Table 4:** Administration situation of CPT-11 and PLD

Patient no.	Level	CPT-1l (mg/m^2^)	Stage	Cell type	Total cycles	Delay cycles (day l)	Skip cycles (day l5)	Dose reduction
1	1	50	IIIc	SAC	4	2		
2	1	50	IIIc	SAC	4			
3	1	50	IIIc	SAC	2		1	
4	2	60	IIIc	EM	4	2		CPT-11 (−10mg/m^2^), PLD (−7.5mg/m^2^)
5	2	60	IIIb	SAC	4	1		
6	2	60	IIIc	CCC	4			
7	3	70	IV	SAC	4			
8	3	70	IIc	SAC	4			
9	3	70	Ic	CCC	2	1		CPT-11 (−10mg/m^2^), PLD (−7.5mg/m^2^)
10	4	80	Ic	CCC	4	1		
11	4	80	IIIc	SAC	3		1	CPT-11 (−10mg/m^2^), PLD (−7.5mg/m^2^)
12	4	80	IIIc	SAC	4			

*SAC* serous adenocarcinoma, *EMC* endometrioid adenocarcinoma, *CCC* clear cell carcinoma, *CR* complete response, *PR* partial response, *SD* stable disease, *PD* progressive disease

CPT-11 on day 15 was skipped in one patient each at level 1 and level 4, with the rate of skipping this treatment being 4.7 %. In the patient at level 1, CPT-11 administration in the second course was postponed because the neutrophil count on day 15 did not meet the criterion for administration, and the study was terminated during the second course at the discretion of the attending physician. In the patient at level 4, CPT-11 administration on day 15 in the third course was postponed, and due to the lack of recovery from leukopenia, the study was terminated at the discretion of the attending physician.

CPT-11 and PLD doses were reduced in one patient each at levels 2, 3, and 4 because of grade 4 neutropenia in the previous course. The doses were reduced in the second course in the patients at levels 2 and 3, and in the third course in the patient at level 4.

### Determination of recommended dose

Three patients were assigned to each dose level, and none of them experienced DLT during the first course, precluding the determination of MTD. Therefore, the individual cases were analyzed in detail. At level 4, grade 4 leukopenia was observed in two of three patients and grade 2 leukopenia in 1. The antitumor effect at level 4 was a PR in two of the three patients and stable disease (SD) in 1. The patient with SD had clear cell adenocarcinoma. At level 3, PR was observed in one of the three patients and SD in 2. As regards treatment postponement at level 4, CPT-11 administration on day 1 was postponed in the fourth course in 1 patient, and day-1 administration in the second course and day-15 administration in the third course were postponed in 1 patient (treatment in this patient was terminated due to the lack of recovery from adverse reactions). Thus, postponement of the next course occurred in two of the three patients. In addition, in one patient at level 4, CPT-11 and PLD doses were reduced in the third course because of grade 4 neutropenia in the previous course. These results suggested that more severe neutropenia would occur if CPT-11 is increased to level 5 (90 mg), although a greater antitumor effect could be achieved. Also, it was expected that the start of the next course would be postponed, and the dose would have to be reduced in a greater number of patients. Based on the above considerations, it was concluded that the recommended CPT-11 dose should be 80 mg/m^2^.

### Antitumor effect

Table 5 shows antitumor effects at each level. CR was observed in one patient (8.3 %), PR in six (50.0 %), SD in two (16.7 %), and PD in three (25.0 %), with the response rate being 58.3 % and the disease control rate 75.0 %.

**Table 5:** Treatment response

	CR	PR	SD	PD	Overall response (%)
Level 1 (*n*=3)	1	1	0	1	66.7
Level 2 (*n*=3)	0	2	1	0	66.7
Level 3 (*n*=3)	0	1	0	2	33.3
Level 4 (*n*=3)	0	2	1	0	66.7
Total (*n*=12)	1	6	2	3	58.3

*CR* complete response, *PR* partial response, *SD* stable disease, *PD* progressive disease

## Discussion

The combined use of the topo-I inhibitor CPT-11 and the topo-II inhibitor PLD is expected to be effective, as suggested by their synergistic mechanisms of action. Also, this combination therapy allows dose reductions in each drug as compared with the monotherapy doses, thereby reducing the severity and frequency of adverse events without decreasing the antitumor effect. In light of the above, CPT-11/PLD is expected to be effective against recurrent or advanced ovarian cancer resistant to platinum or taxane agents. In the phase II clinical study on CPT-11 50 mg/m^2^ (days 1, 8, 15) and doxorubicin (DXR) 40 mg/m^2^ (day 3) combination therapy for recurrent ovarian cancer, Nishimura et al. [11] reported that the response rate was 23.5 % (CR, 1 patient; PR, 3 patients of 17 patients) and that the grade 3/4 adverse reactions observed were neutropenia (CPT-11, 52.9 %; DXR, 35.2 %), thrombocytopenia (5.9 %, 17.6 %), anemia (17.6 %, 0 %), and diarrhea (5.9 %, 0 %). In the present study, the rates of skipping CPT-11 on days 8 and 15 were 6.0 and 22.0 %, respectively, and that of DXR on day 3 was 4.0 %. In the phase II study on CPT-11/etoposide combination therapy for recurrent small-cell lung carcinoma, Masuda et al. [12] reported that 6 of 25 patients (24 %) received two doses of CPT-11(days 1 and 8 or days 1 or 15), and 3 of 25 patients (12 %) could receive only one dose of CPT-11. By referring to the results of the two aforementioned phase II clinical studies, we designed an administration schedule for CPT-11/PLD combination therapy. If CPT-11 was to be administered on a weekly basis, it was anticipated that the rates of skipping on days 8 and 15 would be high, resulting in 30–50 % of patients skipping day 15 administration. Therefore, it was decided to administer CPT-11 on a biweekly basis (day 1, day 15). Also, it was reported that, in giving combination therapy with a topo-I inhibiter and a topo-II inhibiter, these agents act competitively rather than synergistically if administered simultaneously [13]. By also taking account of the finding that CPT-11 is metabolized in 72 h [14], it was decided that PLD would be administered on day 3, as has been the case with combination therapy employing DXR. In past reports, the dose of PLD in the combination therapy for recurrent ovarian cancer was 25–30 mg/m^2^ [15, 16]. While the recommended dose for PLD monotherapy is 50 mg/m^2^, sufficient efficacy and reduced adverse reactions have also been reported at the dose of 40 mg/m^2^ [17]. Therefore, in consideration of reduced adverse reactions such as hand-and-foot syndrome and mucositis, the PLD dose was fixed at 30 mg/m^2^. As to CPT-11, no clinical study results are available for the combination with PLD or doxorubicin by the biweekly method and its optimal dose is unknown, and enormous variation in the dose response of CPT-11 among individuals, in general, is known [18]; therefore, the dose of CPT-11 alone was increased. A phase I clinical study was planned to investigate the efficacy and safety of CPT-11/PLD combination therapy for recurrent ovarian cancer.

Adverse events were evaluated for each patient in the first course only. As shown in Table 3, although adverse events were observed, all were nonserious and manageable. Thus, the phase I clinical study could be conducted safely. Shoji et al. [19] reported that the rate of skipping CPT-11 on day 15 was 2.3 % with the combination therapy based on CPT-11 (60 mg/m^2^) biweekly administration with etoposide oral administration for recurrent ovarian cancer. In our present study as well, CPT-11 was administered on a biweekly basis (days 1, 15), and as a result, day 15 administration was skipped in only 1 patient each at level 1 and level 4. Thus, CPT-11 on day 15 was skipped in only 2 of a total of 43 courses, with the rate of skipping treatment being just 4.7 %, suggesting biweekly administration of CPT-11 in CPT-11/PLD combination therapy to be appropriate. Since no DLT was observed at any of the levels tested, it would be feasible to increase the dose of CPT-11 to level 5 under ordinary circumstances. However, as mentioned above, as a result of detailed evaluation of grade 4 neutropenia in three patients at level 4, postponement to the next course, dose reduction, and antitumor effect, the recommended CPT-11 dose was determined to be 80 mg/m^2^.

The response rate to combination therapy using CPT-11 or PLD for platinum-resistant recurrent ovarian cancer is 41.9 % with CPT-11/VP16 therapy according to Shoji et al. [19] and 44.4 % according to Nishio et al. [20], and 20 % with DTX/CPT-11 therapy according to Polyzos et al. [21]. The response rate was also reported to be 22 % with PLD/Gemcitabine therapy by Skarlos et al. [22] and 40 % [23] by Mirza et al. The response rate to CPT-11/PLD combination therapy was 58.3 %, a result not inferior to those reported previously. All 12 enrolled patients had previously been treated with taxane or platinum agents. In particular, eight of them had recurrence or recrudescence within 6 months after TC therapy. It is expected that CPT-11/PLD combination therapy will achieve a high response rate and that adverse reactions will be mild and manageable. This drug combination may thus be useful as an option for second-line chemotherapy for recurrent ovarian cancer within 6 months after TC therapy.

In the future, a phase II clinical study will be conducted to validate the usefulness of CPT-11/PLD combination therapy.
